# Space, time, and presence in video consultations: an interview study in Danish general practice

**DOI:** 10.1186/s12875-024-02660-6

**Published:** 2024-12-19

**Authors:** Frida Greek Kofod, Anne-Marie Søndergaard Christensen, Elisabeth Assing Hvidt, Anne Beiter Arreskov, Ann Dorrit Guassora

**Affiliations:** 1 The Research Unit and Section of General Practice, Department of Public Health, University of Copenhagen, Copenhagen, Denmark; 2 Centre for Philosophy of Health, Department of Design, Media and Educational Science, University of Southern Denmark, Odense, Denmark; 3 Department of Public Health, SDU Research Unit of General Practice, University of Southern Denmark, Odense, Denmark

**Keywords:** Time, Space, Presence, Doctor-patient interaction, Video consultation, General practice, K.E. Løgstrup

## Abstract

**Background:**

The purpose of this interview study was to explore patients’ and general practitioners’ (GPs’) experiences of space, time, and presence in video consultation in general practice in Denmark.

**Methods:**

The study included six GPs and seven patients from the Copenhagen area, with different experience of video consultations. The data consisted of semi-structured interviews with all participants including recordings from their video consultations. The transcribed interviews were analyzed by Interpretative Phenomenological Analysis (IPA). The theoretical analysis was inspired by philosopher K.E. Løgstrup’s writings about time, space, presence and sensation.

**Results:**

Both the patients and the GPs experienced a lack, or a different form, of presence in video consultations, comparing it to face-to-face consultations. Patients felt more secure in their own homes and the GPs found some of them to be more relaxed during the video consultation than in the face-to-face consultation taking place in the surgery. However, the consultation felt more superficial, with the GPs and patients experiencing an alteration in their sensory access to one another. The video consultation was also perceived as purpose-driven and action-oriented.

Both patients and GPs felt that time was saved. According to K.E. Løgstrup, our experience is always composed of spatiality and temporality; the space is where we sense one another and experience duration, while time is the awareness of change and action. The theoretical analysis points to the experience of presence as spatial and, owing to the changed space in video consultations, the experience of presence and time is changed.

**Conclusion and Implications:**

The balance between space and time is altered in the video consultation. GPs and patients lack certain sensory impressions, owing to the changed spatiality. The changed spatiality, sensation and experiences of presence lead the participants to eliminate the expendable elements to make the consultation more efficient.

Video consultations allow some issues to be handled quickly, but the option for physical consultations still needs to be available, as we believe we now can argue that the physical consultation room has importance for the experience of presence and time.

**Supplementary Information:**

The online version contains supplementary material available at 10.1186/s12875-024-02660-6.

## Background

The consultation room is the place where the patient and the doctor meet. The patient, with their current, past or future illness, symptom, or fear; and the doctor, with their experience, knowledge and task. They come together at an agreed time and often for a predetermined purpose – a consultation. Traditionally, the room where the patient and doctor meet is located in the doctor’s clinic. However, it has not always been this way, nor will it remain so.

Armstrong [1] examines the spatial and temporal history of consultations in “Space and Time in British General Practice”. Before World War II, consultations took place in the doctor’s home – in a dedicated room or in his office. Patients would sit and wait in the courtyard, sometimes for half a day. The doctor had no boundary between work hours and leisure time. He had a specific role in the town and would also make house calls; illness being connected to the domestic sphere. In the post-war years, a different form of consultation space began to emerge. Clinics were formed, with waiting rooms, consultation rooms, examination rooms, laboratories etc. With multiple doctors in the clinic, the need for notes on consultations arose. When notes turned into medical records, a narrative and a timeline of illness emerged for each patient. Illness shifted from an acute event to a process, now possibly becoming chronic and preventable. Illness transformed from being a *spatial* event in the body at home to a *temporal* one. The consultation room had become an institution, perhaps far from the doctor’s residence, and now the doctor’s time was separated into work hours and leisure time (ibid.). The “GP” became the “clinic”, where illness could also be prevented and controlled, with an aim to *save the future*.

Several researchers have argued that the design of the consultation room plays a role in shaping a consultation. Almquist et al. [2] describe how the design of the consultation room impacts the interaction between individuals — the interpersonal-space interaction Ajiboye et al. [3] found the *communication* between doctors and patients affected by different designs. Most notably, there were significant differences across clinic designs in patients’ ability to view the computer screen displaying electronic medical records when desired and to search the internet together with the doctor. This had implications for patients’ engagement with their medical records, information sharing and shared decision-making [2, 3].

There has been a significant focus on the concept of time in general practice since the post-war years [1] and temporality has remained a topic of interest. Even back then, GPs had limited time — a situation which persists today [4, 5]. In 1953 Armstrong described a picture of the “modern waiting room” as a “bright, clean space radiating efficiency” [1], where efficiency was regarded positively because it allowed better resource utilization per unit of time. The pursuit of efficiency is thus not a new phenomenon. Time is scarce and we strive to use it as effectively as possible, filling it with meaningful work, expending minimal effort and resources and achieving the same or better results, all within the shortest possible timeframe [6].

In the quest for an efficient consultation type that optimizes time, many healthcare providers have implemented video consultations (VCs) as a solution [7–9]. This development was supported by a pandemic that made physical meetings impossible, as well as by regional authorities. VCs involve the patient and the healthcare provider being in separate locations, interacting via a camera on their computer, tablet or smartphone through an app [10, 11]. In Denmark, both GPs and patients use the “Min Læge” (My Doctor) app [12]. During VCs, patients and doctors can converse synchronously, and each can see the other’s image. The consultation room has become virtual, with participants spatially separated. While doctors work from their clinics, patients have the freedom to choose their location — often their own homes or the workplace. The physical space is no longer shared: the participants occupy separate rooms and are subject to different sensory impressions from their surroundings [2, 13, 14].

Before the internet, experiences of space, spatiality and presence of sensing individuals in the same space were not separate. Sociologist Erwin Goffman defined copresence as two bodies in the same room [15, 16]. However, with the emergence of the internet and the possibility of remote interaction a new explanatory model for presence had to be developed. In his description of the phenomenology of perception Shengli [17] argues that Merleau-Ponty’s conception of space provides an opportunity to understand the experience of space without assuming the physical presence of two bodies. Instead, the experience of space is understood as a “form of perception”. According to Merleau-Ponty, this perception is “the universal power enabling them (i.e. things) to be connected” (ibid.). In other words, he opens the possibility that physical space is not necessary for the experience of space and presence.

However, the experience of space is different in VCs, as not all senses are stimulated [14]. In our previous study [18], we found that GPs experienced altered sensory conditions when interacting with patients during VCs. This, combined with Almquist’s [2] findings — where the design of the physical consultation room affects the doctor-patient interaction — raises questions about how the consultation experience is influenced by the consultation room being virtual. In this virtual kind of room, a physical examination by the doctor is not possible, the visual field is limited and the senses of touch and smell are excluded [14]. Historically, changes in the framework of consultations have also impacted the perception of time [1]. The question is whether the perception of time is also altered during VCs.

Our aim in this study is to investigate how space, time, and presence are experienced by patients and GPs during VCs.

## Methods

### Setting

In Denmark, the GP service agreement was expanded during the COVID-19 pandemic in 2020 to include VCs. The present study was conducted in a Danish general practice setting after the lockdown in 2021–2022 when patients again had physical access to their doctors. Consultations in Danish general practice are funded and free at the point of use [19]. At the time of data collection, the GP’s fee for a face-to-face consultation was slightly lower than for a VC.

### Participants

Six GPs were participating. They were recruited consecutively by e-mail and telephone. 70 were contacted: 59 from the list of GPs in the Capital Region. Every 5th clinic was contacted if they offered VC according to their web page. 11 were from network or had participated in earlier studies by some of the authors. Several GPs felt pressured, during period, due to factors such as the inclusion of COVID-19 vaccination, leading them to decline to participate. There was good variation among the participating GPs in their practice experience, and experience with VCs (see Table 1).

**Table 1:** The participating GPs

Participants				
General practitioner	Age (Years)	Sex (Male/Female)	Experience as GP (Years)	Experience with video consultation
Laura	40–49	Female	5–10	Minor
Bridget	50–59	Female	10–15	Moderate
Donald	60–69	Male	25–30	Moderate
Brian	40–49	Male	10–15	Extensive
Sarah	40–49	Female	0–5	Moderate
Pete	50–59	Male	10–15	Extensive

The GPs were asked to record at least one VC for use in the study. All Danish-speaking patients (adults without dementia or psychosis) who attended a VC were eligible. Seven patients were included by the GPs (see Table 2).

**Table 2:** The participating patients

Participants				
Patient	Age (Years)	Sex (Male/Female)	Occupation	Experience with Technology
Lisa	17–29 ^a^	Female	High school student	Moderate/extensive
Wendy	40–49	Female	Office worker	Moderate
Patrick	30–39	Male	Freight forwarding agent	Extensive
Sam	30–39	Male	Chartered surveyor	Extensive
Cathrine	17–29 ^a^	Female	Student to become qualified social worker	Moderate
Dan	70–79	Male	Retired sales manager	Moderate
Patricia	40–49	Female	Self-employed	Moderate

^a^We got consent from a parent when the patient was 17–18 years old, but the parent did not participate in the consultation nor interview

The consultations addressed health problems such as rash, aerophobia, depression, eating disorders, regulation of medicine or annual monitoring of blood pressure. Two of the consultations were about new problems and five were follow-ups. The duration of the consultations ranged from five to 16 min (see Table 3).

**Table 3:** An overview of the consultations

Consultation				
Patients	General practitioner	Duration	Subject	Patient’s location during the consultation
Lisa	Laura	16 min	Mental health and everyday function/eating disorder?	At home, sitting in bed
Wendy	Bridget	10 min	Blood pressure	Office at work
Patrick	Donald	11 min	Treatment for depression and asthma	At home, in a home office? A neutral place with a white background
Sam	Brian	7 min	Rash (= new)	Workshed, alone
Cathrine	Sarah	15 min	Eating disorder/depression	Home, on the sofa, in a dressing gown
Dan	Pete	10 min	Annual control for blood pressure	In summer residence
Patricia	Pete	5 min	Aerophobia (= new)	At home, on a chair in living room

### Design and data generation

Many patients and GPs spontaneously compared VCs to face-to-face consultations in the semi-structured interviews of this study. While some follow-up questions may have prompted comparisons or sought clarification when participants found experience with VCs difficult to explicate, the interview guide (supplemental files 1 and 2) primarily consisted of open-ended questions without comparison to face-to-face consultations. The guide was focusing on the interpersonal contact between patients and GPs along with their experiences with communication in VCs and of not being in the same room. This approach allowed for natural emergence of comparative experiences. Toward the end of each interview, the participant and the interviewer (FGK) watched the recording of the VC. With inspiration from Video Stimulated Interview technique (VSI) [20], the interviewer asked supplemental questions, relating to eye contact, presence, body language, verbal repetitions, interruptions, etc. Interviews were recorded and transcribed verbatim.

Five of the interviews with the GPs took place in the GPs’ surgeries, one at the Centre for General Practice, as well as one with the patients. Two of the interviews with the patients took place online, using a safe Zoom-link, and four took place in the homes of the patients.

The interviews were 42–72 min long and were conducted within two weeks after the VC had taken place. The quality of the interview dialogue with participants was strong with a comfortable interviewer who succeeded in creating the confidence needed for obtaining valuable information, and mostly articulate participants [21]. Both the patients and the GPs were aware that the interviewer was a doctor. She was aware of her position as a colleague to the GPs and ensured that they knew she was not evaluating their work, but rather exploring their experience of the VC. The patients were reassured that the interview did not influence their treatment and that the GPs did not receive any information about the patients from the interviewer. An analysis with a different aim has previously been carried out on the GP interviews and published [18].

### Analysis

The interviews were analyzed using Interpretative Phenomenological Analysis (IPA) [22], which is suitable for exploring first-person experience and subjective meaning-making. The first author read all transcripts repeatedly and made exploratory notes focusing on the perceptions of the participants, and their experiences with interpersonal contact and communication in VCs. These notes were compared with exploratory notes made by the last author for six interviews. Experiential Statements were written and clustered as Personal Experiential Themes (PETs) for each participant (ibid.). Group Experiential Themes (GETs) were subsequently created across patients and GPs, separately, with specific interest in themes across the data, or themes strongly represented in one of the groups of participants (patients or GPs) (ibid.). The process was iterative, moving between themes and individual interviews. All authors read at least two transcripts and contributed to a discussion of PETs and GETs. The first author drafted the manuscript.

The preliminary analysis revealed that both space and time were important themes for the patients and the GPs. We focused on these aspects in the analysis and explored connections between them.

As a specialist doctor in family medicine, the closeness to the patient in a face-to-face consultation is highly valued by the first author. She is therefore curious about how to achieve good intersubjectivity and relational quality in VCs. Through the processes collection and analysis, she was aware of her preconceptions and the importance of maintaining openness and curiosity. Her preconceptions were also challenged in discussions with the interdisciplinary team of authors and a research network on VCs.

## Results

The results can be summarized as follows. Both the patients and the GPs experienced a lack, or a different form of, presence, in VCs, when they compared it to face-to-face consultations. The patients felt more secure in their own homes and the GPs found some of the patients to be more relaxed during the VC, than in the face-to-face consultation taking place in the surgery. Nevertheless, the VCs were experienced as more superficial, with the GPs and the patients observing a reduced sensation of one another, not being in the same room. The conversation was experienced as simpler and more concrete, and the consultation as more purpose-driven and action-oriented on video than face-to-face. The patients and the GPs felt that time was saved.

### Feeling secure in the home environment but superficial

In a VC, the patient is often at home or at work, while the doctor is in her/his familiar consultation room in the clinic. For some patients, it was easier to talk to the doctor in a video consultation than face-to-face, since being at home supplied energy for the conversation and made them feel secure. One patient experienced the physical distance that the VC room provides as protective; she felt less exposed and was able to remove herself quickly from a shameful situation. This may be an expression of the patients feeling more secure in the room at home.

Several of the doctors perceived the patients to be more relaxed in their own homes. Laura, a GP with many years of experience, but who had only conducted a few VCs, shared the following:

I quite like this, you know. It’s that she becomes more natural in her home environment. She’s willing to share more. Down here she’s much quieter. Somehow, she sits there and talks, glancing up at the ceiling, pondering. She truly feels at ease in her own home. So, I believe I gained much more insight from her during this interaction than when she was up here. It was rather intriguing.

When patients were in their homes during VCs, GPs perceived that insight into the home environment could enhance their understanding of the patient, despite a limited view of the patient on the screen. The GP is “visiting the patient’s living room” and one GP described the patient and the doctor as both being “on home turf”, rather than just the doctor, as is the case in physical consultations.

Despite the homely setting, patients typically did not talk about deep subjects with the doctor when interacting in video consultations, and several experienced that the consultation became superficial. Cathrine, a young patient, described it like this:

Video consultations can tend to become superficial—you know, where you just quickly respond with yes or no because it’s a bit fast and easy to get through. And, of course, during follow-ups, that’s not a problem. But when you need to give your full self to something, whether it’s starting a treatment plan or—regardless of where it takes place—you should ideally feel that you can give everything of yourself.

Several patients also claimed that technical delays and difficulties in interpreting body language contributed to making the conversation more superficial. Similarly, the GPs experienced that sensitive topics were discussed less in VCs than in face-to-face consultations.

### Experience of less presence in the VC room

The sensory conditions and the sensation of one another were altered in the VC room, compared to the physical room. For some patients, the video meeting felt “empty”: they could not be offered a cup of coffee or a tissue to dry their tears, and they could not fully immerse themselves in the experience or sense the other as a human being. There was an experience of diminished presence. An elderly gentleman, Dan, who went to his doctor for a blood pressure check, claimed that he sensed other people better in the physical room than in the VC room. He said:

…He can also read me better, and I can read him better too… We must also be careful not to… drift apart in a way - that it’s [the meeting is] not just on video, or when we call or something similar… Isn’t it a bit empty, or what should I say? I don’t have the exact word, but empty, if we don’t occasionally see the person.

Some patients described that the presence *is not there* in VCs, due to the absence of physical presence and eye contact. On the other hand, some patients experienced that doctors were present somehow by being attentive — listening, asking questions, and taking their time.

The patients’ experience of diminished presence in the VC room than in the physical room was often shared by the GPs. The GPs perceived that reduced sensation of one another in the VC room became a barrier to achieving a togetherness or having that close space with the patient, as in the physical room, in which they sensed one another. Donald, a highly experienced GP, explained:

I just don’t feel it comes across. I mean… I also don’t really think I can give as much there [on video]. Because, you know, I can’t sit there and talk about magic and other peculiar things when there’s a screen between us… Because: that shared understanding of having that very close space, where you can sort of sense each other and all that, right? I feel like you just don’t have that on video.

When the GPs, as part of the interview, watched themselves in the recordings of their video consultation, some saw how they were occasionally looking away in the VC, taking this to mean that they had been absent towards the patient. They compared this with the interaction taking place in the physical room, where altered eye contact was not perceived as absence in the same way. A common thread among all participants was that they experienced better interpersonal contact and trust when they already knew each other from the physical room. Donald expressed this as follows:

…we had established that connection many years ago, right? So, it was good support for her to just say hello, ask how things are going with you, and now I’m just checking in, right? So when I think of her—that worked well, but it’s also because I know her, and we just need to say something—a word, and we know what the other person is thinking, so….

Familiarity with the patient allowed GPs to recognize reaction patterns and facial expressions, and to gauge their level of concern.

### Time becomes prominent in the VC

Both the GPs and the patients perceived VCs as quick, efficient, and focused. Several patients mentioned that they usually had an issue to clarify, and the conversation tended to get straight to the point. They were able to check off items, get things fixed, and *just click*. One patient described getting what he came for and then moving on.

For doctors, consultations in the virtual room were action-oriented and purpose-driven. They quickly got to the heart of the problem, avoiding or missing out on any small talk. Donald explained:

…So, it becomes a bit more concise and focused when you’re sitting in front of a screen… Similar to phone calls… there’s a messaging function, right? You have a message to deliver, the purpose of this call, and that’s it. So, we have an agreement.

The patient, Sam, believed that one must accept that the doctor cannot assess everything during a quick video appointment. Sarah, with only a few years of experience as a GP, experienced that the pace of the VC could lead to misunderstandings. Perhaps the doctor would miss that the patient had not fully grasped the topic due to the rapid pace. She said:

But that’s how the video format might work—seems a bit faster. ‘Yes, yes, it’s fine, yes, we got it, like boom boom boom,’ and you don’t realize that the patient hasn’t understood what it’s supposed to be about, or ‘now we’re changing the dosage’ or something like that, right?

She also observed that patients might hesitate to ask for more when things needed to move quickly. One GP said that there was a loss of quality in VC, comparing it with face-to-face consultations where it was easier to establish a calm atmosphere that invited the patient to sit down and to talk.

### Effective frameworks for VCs

Patients experienced saving time when they saw their doctor via video. Many had not taken time off work but were able to consult the GP from their office for about ten minutes. They found it easy and straightforward to obtain an appointment at short notice, the consultation was quick, and they were able to return to work promptly after the consultation. Additionally, they perceived it as efficient and time-saving to use the waiting time for other productive tasks. Patrick, a young family man and freight forwarding agent, explained:

It’s both the distance and the time. I’ve lived in [a suburb of Copenhagen] for many years, and it wasn’t far away, but you still save time… It’s easier for me because I can log in, run around doing other things, and then hear a ‘ping,’ now Donald is there, and I can grab a chair and have the conversation with Donald.

Like Patrick, many patients appreciated saving time and avoiding traveling to the doctor.

GPs experienced that VCs could release some time, including providing a little “breathing space” (i.e. unplanned time) in a busy schedule, as they could conduct some consultations more quickly. The GP Sarah found that the consultation went faster because there were no additional concerns or “door knob” questions:

…often, you can make it shorter than that quarter-hour, you could say. So—it also became a buffer for me in a busy schedule, where sometimes you could save five minutes because you can often handle it a bit faster, and… for some reason, patients don’t have all those ‘door knob’ questions or those ‘while I’m here, I also have…’.

Most of the GPs related that both doctors and patients expected a brief and fast consultation, and ideally the consultation should not develop into something requiring a physical examination.

## Theoretical analysis

### Spatiality withdraws and temporality emerges

In our empirical data, we found that time was brought to the fore in VCs, which both doctors and patients perceived as quick, efficient and focused. The conversation went straight to the point, and only one specific issue was discussed. There was also a reduced sensation and sense of presence in the VC room.

K.E. Løgstrup develops a conception of our experience as fundamentally shaped by time and irreversibility on the one hand, and space and sensation on the other. Our perception of time is connected to change and efficiency, while our experience of space is linked to sensation, presence and closeness. We cannot experience anything without time and space, so temporality and spatiality are always involved in our experiences. Space understood as spatiality is a precondition for our perception of time and duration. Space as a timeless presence, in fact, enables a transcendence of the passage of time. Løgstrup also points out that our experience of space and time is interconnected. When time and change come to the forefront, space and presence recede into the background, and vice versa [23].

In VCs, the consultation room has changed, and the sensory conditions are altered. The doctor and patient sit in separate rooms, with a digital space between them, unable to perceive each other fully with all their senses — the way we typically experience presence, according to Løgstrup [23]. Our findings revealed that both doctors and patients perceived reduced presence in the video-mediated consultation room, which sometimes felt empty and superficial. The sensation and trust between doctor and patient are often established beforehand, in the physical room. However, in VCs spatiality, presence, and sensation become impoverished—they take a step back. This means that one is less present in the room and more focused on the temporal now, directed toward the future.

Løgstrup describes how time and temporality are brought to the fore when we plan and act, when we believe things should be fast, efficient and focused. In our data, this manifested when participants in the consultation: stuck to the topic, discussed one topic only, were purposeful and in motion, kept the conversation on point, and felt the need to “move on”. The consultation was perceived as shorter and participants felt that they saved time, including travel time. Sometimes the GP’s schedule even gained “more breathing room.” There was also an economization of words. Communication became simpler and more concrete. Time emerged.

The connection between the experience of time and space, as established by Løgstrup, supports an interpretation where the reduced spatiality, sensation and presence — found in our data — contribute to bringing time to the fore in VCs. The participants eliminate the expendable elements, making the consultation more efficient.

## Discussion

Patients and GPs, in this study, perceived diminished presence in the VCs, compared to the experience in face-to-face consultations, reflecting altered spatiality and sensation. Yet patients often felt that GPs were present in some way during the VCs. This suggests a different form of presence, as we will discuss first. VCs can be experienced as more relaxed than face-to-face consultations, with both parties on familiar ground. Even though the patients are more at ease, they may avoid talking about deeper issues with the doctor in these consultations. This apparent paradoxicality will be discussed next. Finally, we will discuss time and space in the context of consultations. As argued above drawing on Løgstrup´s writings; when space recedes, time is brought to the fore, and the VC becomes purpose-driven and efficient.

### Presence and space

In our findings, the GPs experienced the reduced sensory conditions of VCs, compared to face-to-face consultations, as a barrier for achieving togetherness with patients and having this close space, where they could fully engage and discuss deeper matters. Both doctors and patients perceived the consultation as more superficial, with diminished presence during the consultation.

On the other hand, the patients in our study experienced some kind of presence, especially when the GPs showed concern and inquired further about their issues. This could be an expression of a different form of presence or spatiality in the digital space, as introduced by Lindemann et al. [16], according to whom physical space is not a prerequisite for the experience of presence. Oudshoorn [9] also describes in her study that digital presence can be established between nurses and patients, but it requires multiple calls and adjustments from the nurses.

However, if the encounter in a VC does not occur in a physical space but exists in time, as Zhao [15] suggests — where individuals “share a community of time without sharing a community of physical space” (ibid.) — then an actual physical room is not required. This argument could imply that the need for physical space is removed from the human encounter and the experience of presence.

Nevertheless, space appears to make a difference. This is evident both in terms of what the physical design of the room does for the consultation and communication, as described by Almquist et al. [2] and Ajiboye et al. [3], and in our data, where the sensation within the video-mediated room — i.e. the sensory experience — is different. The room has receded, to use Løgstrup’s description [23].

The fact that both GPs and patients, in our study, experienced a lack of presence while still perceiving some form of presence opens up new understandings of what presence can be. In our data, we observed that GPs, when reviewing the recording of the video consultation, quickly felt absent when they momentarily looked away from the screen/camera. To maintain contact with the patient, the nature of the video room created a need for them to repeatedly recreate the experience of presence. There was an ongoing recreation of presence. In this consideration, we might draw upon Shengli’s [17] description of presence as a perception, where the experience of space does not necessarily require the physical co-presence of two bodies. If the experience of presence needs to be continually reproduced, it also suggests that time plays a role in this context — that time influences the experience of presence. When space as sensorial presence recedes then mutual presence has to be established in time, and in time presence has to be continuously recreated.

### At Home, but not Profound

Our results revealed that from the GPs´ perspective, some patients were perceived to be relaxed during VCs, especially when they were consulting from their own homes. Despite this, they preferred to not discuss deeper matters with the GP in these consultations. Additionally, non-verbal cues and sensory impressions from the non-verbal communication between our participants were also altered in the VC room. This aligns with what Kim et al. [24] describe, about non-verbal communication influencing the degree of intimacy and immediacy (which includes psychological distance or presence) (ibid.). Therefore, the modified body language, or altered perception of body language, in VCs, may be one of the reasons why patients avoid discussing deeper issues with the doctor during VCs.

### Time, space and the consultation

In our theoretical analysis, we explored how time manifests, and is brought to the fore in VCs. This perspective is novel compared to the existing literature, which primarily focuses on the time-saving aspect of VCs [8, 25, 26]. In research on face-to-face consultations in general practice Davidsen and Reventlow [27] discuss the concept of time. They note that therapeutic consultations create a different rhythm of communication, where inner time is experienced differently. Doctors and patients enter a sort of “timelessness” during the therapeutic conversation, where time recedes, and space is brought to the fore. However, the opposite seems to occur in VCs, according to our findings. Davidsen and Reventlow also found that doctors inquire less about emotional matters and adopt a more biomedical approach when pressed for time [27]. These findings align with our results, where communication becomes more focused and targeted when time is brought to the fore. It is also in line with what doctors in our study experienced — that the rhythm of communication is different.

We can argue that the balance between time and space is altered in VCs. Reduced spatiality implies that there is much less to anchor time (to give the situation duration), as we lack certain sensory impressions. The consequence of altered sensation and spatiality is increased efficiency. According to our previous study [18], GPs take this into account when selecting topics for VCs.

### Strengths and limitations

One of the strengths of this study is that interviews with patients and GPs are based on specific VCs, which are shown and reviewed as part of the interview. It made participants aware of the crucial role of eye-contact and non-verbal communication in creating a sense of presence during interactions. Despite that several participants also spoke about their experiences in general, we believe it is a strength that the patient and the GP encountered and reviewed the same consultation as it allowed us to combine the different perspectives in the analysis.

Another strength is that among GPs and patients a variety of attitudes towards technology and VCs were represented. This means that the study findings can be broadly applied to contexts where GPs and patients have just started conducting VCs, as well as situations where they have extensive experience with them. Furthermore, the study was conducted post-COVID-19, when society had fully reopened after the pandemic, and we believe this strengthens the usability of the results in general practice today.

The interdisciplinary team of authors with different theoretical backgrounds is another strength of this study. All the authors contributed to a broad insight into the discussion of data analysis. The first author, who conducted all the interviews, is herself a trained specialist in family medicine. Therefore, she was familiar with the setting of the GP-patient consultation and did not need to spend time getting to know that setting. At the same time, she remained curious and discussed her preconceptions with the co-authors and research colleagues.

In Denmark, patients do not have to pay to see a doctor, meaning that everyone, regardless of income, has access to their GP. This implies that our study cannot be directly transferred to healthcare systems with a different financing model. Further, in this study, the patients and the GP knew one another from before. We know from research that beforehand knowledge affects the experience of the VC [28], so the study may not be directly transferable to contexts where the GP and patient are not familiar with each other.

The study describes conditions in an urban area with relatively short travel distances from patient to doctor. We do not know if patients’ preferences for physical visits to the GP would have been different in areas where transportation distances are greater. There may have been an underrepresentation of certain groups in this study, limiting the transferability. Research shows that VCs, compared to face-to-face consultations, disproportionately attract younger patients, potentially underrepresenting older individuals [29].

### Conclusion and implications

This study shows that the experience of presence is changed in the VC room. This alteration is due to the reality of physical distance and the virtual mediation of the VCs: space is altered in VC and does not enable the same sense of presence and sensory conditions as face-to-face consultation. Participants eliminate the expendable elements in VC, making the consultation more efficient.

VCs allow some issues to be handled quickly, but the option for physical consultations still needs to be available, as we believe we now can argue that the physical consultation room has importance for the experience of presence and time.

## Supplementary Information

Supplementary Material 1

Supplementary Material 2

## Data Availability

The transcribed anonymized interviews used for this study are available in Danish from the corresponding author on reasonable request.

## Abbreviations

GP: General practitioner
IPA: Interpretative Phenomenological Analysis
VC: Video consultation
VSI: Video Stimulated Interview
PETs: Personal Experiential Themes
GETs: Group Experiential Themes
