# Supplementary material for: Space, time, and presence in video consultations: an interview study in Danish general practice
Source: BMC Prim Care. 2024 Dec 19;25:425. doi: 10.1186/s12875-024-02660-6 (PMC11656864; doi:10.1186/s12875-024-02660-6)
Supplement: Supplementary file 1 — Supplementary Material 1 [file 12875_2024_2660_MOESM1_ESM.docx]

### Interview guide – interviews with patients

| Process | Themes | Questions | Follow-up questions |
| --- | --- | --- | --- |
| Introduction | Presentation and the framing for the interview | First, we will talk a bit generally, and I will ask some questions about your relationship with your GP, and the connection you have. Then we will talk about the consultation you had on video with him/her last XX-day, and we will watch the recording together, and I will ask some questions, and you are free to stop it and comment.  There is no pressure – you just say what you feel like, and I will ask if I am in doubt of what you mean. Is that ok? There are no right or wrong answers. I am interested in your experience, what you think, feel and say.  I will record the interview, and it is only me and a few other researchers on my team, that will read the interview. Your GP will not get any information about what you have told me. It will take about an hour, and if you regret to participate, you just tell me, and I will erase it all.  Are there any doubts about how it will proceed? |  |
| The interview | The interpersonal contact, Relationship, Therapeutic alliance, collaboration, rapport, empathy? | For how long have you known your GP? Did You have consultations on video before? How many times?  How would you explain your relationship with the GP?  I am interested in how it is to communicate on video – can you please tell me how it was to talk with your GP on video, there last XX-day?  Did you feel listened to? Did you understand each other?  If you have met your GP on video several times; is there anything, you have experienced works better than other things on video? Please elaborate.  Anything that did not work on video? Anything that made you feel it would be better to see the GP at the clinic?  Did you understand the GP? Please elaborate… | - What did the GP do, that made you feel that? - Something he/ she did or said? |
|  |  | Do you do anything differently yourself in terms of body language when you meet in a video consultation? For example, are you more conscious of it?  Could you see the GPs facial expression on video? How was it?  People talk about how it can be a challenge not to see body language when the consultation is on video. Is that anything you have thought about? | Anything the GP did, that made it worse/better? Or can do?  Anything the GP can do to make it matter less or more?  Something else? |
| The interview | Presence, proximity, distance, responsibility, | What is your experience with/How do you feel about that there is a physical distance to the GP? Your experience of not be in the same room as the GP? What does presence or closeness mean to you?  How is the interpersonal contact on video?  Did you feel the GP had the same responsibility in the consultation on video as in a face-to-face consultation?  Is the responsibility for your health shared differently when you meet the GP on video? | Did you feel the GP was present? Please - elaborate |
|  | Video as part of a series consultations | Was the last consultation you had also on video? When was the last time? |  |
|  | See the recording together. | I have seen the recording of the consultation, and we will soon see it together. But first I would like to ask how were you doing during the video consultation and after? Please – elaborate..  Is there anything in this episode that you particularly notice?  In this episode, you say… What do you think about that?  I have noticed that the GP says so, how do you understand that? What is your feeling?  I have noticed that you do XXX/say YYY - what do you mean when you say that? What do you think about that? Feel?  I have noticed the GP says this – what do you think about that? How do you feel about that? Experience? Jeg har lagt mærke til, at lægen siger sådan.. Hvad tænker du om det? Hvordan forstår du det?? Hvad oplever du når hun/han siger/gør sådan? | Do you think it is because of the video format?  When you say, your contact is different, what do you mean by that?  Why is that? Hvorfor tror du at det er sådan?  How do you think it affects your contact, your relationship/contact? |
|  | Therapeutic alliance and contact/rapport, empathy, presence, bond | Do you believe you and the GP had agreed on what the next step was, when you said good bye?  Did you feel safe? Secure?  Did you feel heard?  Do you understand each other? Is there any difference between the understanding during consultation face-to-face and on video?  Did you feel the GP was present? How did you recognize that? | Is it different than if it was face-to-face?  Do you feel the GP is present in an other way on video, compared to when you meet face-to-face? If so, how? |
|  | agenda setting | - How did you figure out, what to talk about? Is it different on video? - Did the consultation deal with what you wished for? Did you talk about what was on your mind? | Was it a different presence?  Were there any topics the GP avoided, due to the video format? What about you? |
| technology |  | - How do you use technology? Is it exciting, helpful, necessary, necessary evil? |  |
| Closing/Round up |  | We are about to conclude.  Is there anything you think I need to know, or that you would like to tell about video consultation and how it affects your contact with the GP?  Is there any question you think was strange, I asked ..? | Why?  Please – elaborate |

Demographic data: age, geography, employment/student/retired, how much experience with video consultation
