# Supplementary material for: Space, time, and presence in video consultations: an interview study in Danish general practice
Source: BMC Prim Care. 2024 Dec 19;25:425. doi: 10.1186/s12875-024-02660-6 (PMC11656864; doi:10.1186/s12875-024-02660-6)
Supplement: Supplementary file 2 — Supplementary Material 2 [file 12875_2024_2660_MOESM2_ESM.docx]

### Interview guide – interviews with General practitioners

| Process | Themes | Question | Follow-up questions |
| --- | --- | --- | --- |
| Introduction | Presentation and the framing for the interview | First, we will talk a bit in general, and afterwards, I will ask some questions about the video consultation you had with XX. Then we will watch the consultation you had with XX the other day, and we talk about it. There is no pressure - you just have to tell what comes to mind - and then I will make sure to ask if I am in doubt. Is that okay?  I am going to record the interview, and it is only me and my supervisors and a few other researchers at the university who will read the interview. If you regret it, you of course have the right to withdraw again. |  |
| The interview | Contact/relation/therapeutic alliance/collaboration/ rapport/empathy(?)/ | Can you please describe your contact with this patient?  What does it mean for the contact with the patient that it takes place on video?  Is there anything you do differently because it is on video?  How do you think the contact works (with this patient) on video?  What works?  Is there anything that does not work?  Did you think it was a good consultation? Why so? What worked well/less well? | - Did you do anything special to build a good atmosphere? Good interpersonal contact? - How did you ensure good interpersonal contact when it is on video? - What is it about video that makes it different to meet? - I think I know what you mean, but I would like to hear in your words how you would explain it if I was not a colleague… How would you, for example, say it to… |
|  | Presence/Proximity/Distance | What is your experience of the fact that the consultations is at distance/that you are not in the same room?  Do you feel obligated in the same way towards the patient when you meet on video?  Does the patient get more power over you? Less?  Do you feel the same responsibility for the patient when you meet on video? | - Please, explain.. |
|  | Video assessment. Contact, presence, closeness, communication.. | How much do you usually see of the patient on the screen? What does it mean compared to a face-to-face consultation?  If something is missing, can it be compensated for? How?  Do you do anything differently yourself in terms of body language when you have a video consultation? For example, are you more conscious of it?  Can you assess the patient’s facial expression on video?  Is there anything that can be done to make it easier?  People talk about how it can be a challenge not to see body language when the consultation is on video. Is that something you have thought about? | Please, elaborate  Do you have an example of how you have done it before? |
|  | See the recording together | What do you think about the eye-contact?/focusing on your contact/  focus on therapeutic alliance. What do you understand by therapeutic alliance?  Is there anything in this episode that you particularly notice?  In this episode, you say… What do you think about that?  I have noticed that the patient says so, how do you understand that?  I have noticed that you do XXX/say YYY - what do you mean when you say that?  Here we can see that you say ZZZ, when the patient has said XX - what do you think about that? | - Do you think it’s because it’s on video? - When you say that your contact is a bit different - what do you mean by that? Why do you think it is like that? - What effect do you think it has on your contact/relationship? - Were there topics you felt you had to avoid because it was on video? |
|  | Therapeutic alliance | If we talk about therapeutic alliance - what do you think of? Do you think you have it?  Is it something you try to achieve? How do you do it? | Is it different on video, than if it was face-to-face? How? |
|  | Agenda setting | How do you figure out what to talk about?  Is it different on video? |  |
|  | To know each other and technology | How long have you known this patient?  Was this consultation part of a series of consultations?  Have you had a consultation on video with this patient before?  What do you think about technology? How do you use it? Is it a help in everyday life? Or some “necessary evil”? |  |
| Closing/Round-up |  | We will soon have to conclude  Is there anything you think I need to know, or that you would like to tell about video consultation and how it affects your contact with patients?  Was there anything strange that I asked about? Something you think is missing? | Why? |

Demographical data: the doctors age, geography, type of clinic, experience as a GP
